# A Numerical Study of the Dynamic Crack Behavior of Brittle Material Induced by Blast Waves

**DOI:** 10.3390/ma16227142

**Published:** 2023-11-13

**Authors:** Haijun Yu, Ming Zou, Jinshan Sun, Yuntao Wang, Meng Wang

**Affiliations:** 1 State Key Laboratory of Precision Blasting, Jianghan University, Wuhan 430056, China; yuhaijun0601@163.com; 2 Hubei Key Laboratory of Blasting Engineering, Jianghan University, Wuhan 430056, China; 3 MOE Key Laboratory of Deep Earth Science and Engineering, College of Architecture and Environment, Sichuan University, Chengdu 610065, China; zouming@stu.scu.edu.cn; 4 Sichuan Shu-Neng Mineral Co., Ltd., Leshan 614600, China; 5 Tianfu Engineering-Oriented Numerical Simulation & Software Innovation Center, Chengdu 610207, China; wangyuntao@scu.edu.cn

**Keywords:** P-wave, S-wave, Rayleigh wave, crack initiation, crack propagation

## Abstract

Blast stress waves profoundly impact engineering structures, exciting and affecting the rupture process in brittle construction materials. A novel numerical model was introduced to investigate the initiation and propagation of cracks subjected to blast stress waves within the borehole-crack configuration. Twelve models were established with different crack lengths to simulate sandstone samples. The influence of crack length on crack initiation and propagation was investigated using those models. The linear equation of state was used to express the relationship between the pressure and density of the material. The major principal stress failure criterion was used to evaluate the failure of elements. A triangular pressure curve was adopted to produce the blast stress wave. The results indicated that the pre-crack length critically influenced the crack initiation and propagation mechanism by analyzing the stress history at the crack tip, crack propagation velocity, and distance. The inducement of a P-wave and S-wave is paramount in models with a short pre-crack. For long pre-crack models, Rayleigh waves significantly contribute to crack propagation.

## 1. Introduction

**Figure 1:**
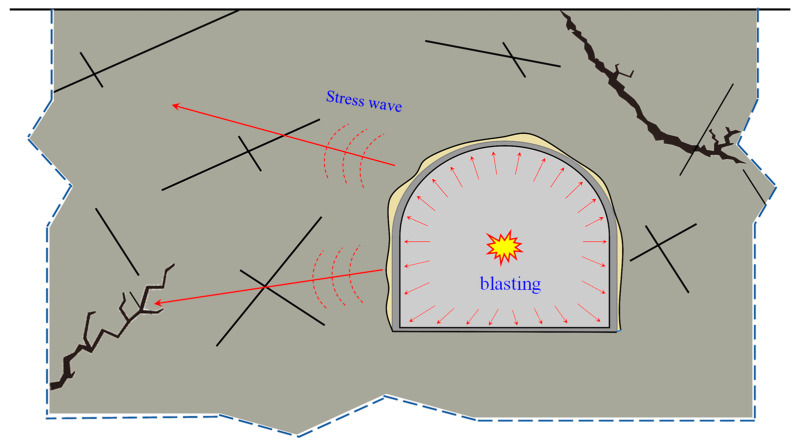
Schematic diagram of fractured rock mass blasting.

The drilling and blasting method [1,2] plays a crucial role in tunneling and underground mining operations, as it effectively governs rock fragmentation, ensuring construction safety and quality [3]. However, the blasting process can notably affect the fragmentation and fracture zones in deep and high-stress environments, which may differ significantly from those observed in conventional conditions. The propagation patterns of blast-induced cracks in multi-hole blasts are characterized by distinct features. The rock mass is characterized by a multitude of pre-existing fractures that have a significant impact on their stability, especially when subjected to high levels of stress. Many of these fractures are in a critically stable state when under controllable conditions. But, during blasting operations, shockwaves are generated, which initiate, expand, and create interconnected networks of fractures in the surrounding rock. Nearby fractures may undergo sudden expansion under stress wave disturbances, which may cause the overall instability of tunnels or underground structures. As such, this series of scenarios can be categorized into two parts: (1) the near-field fragmentation and penetration of porous cracks caused by borehole blasting; and (2) the initiation of surrounding rock fractures induced by stress waves, as depicted in Figure 1. As engineering projects delve deeper into the subsurface, these challenges become progressively more intricate and critical. They necessitate careful consideration in the realm of deep engineering construction.

Explosive loading is acknowledged to manifest in two distinct modes [4]. The initial mode entails the blast stress wave, which triggers the formation of primary networks of fractures within the medium. Conversely, the second mode encompasses the application of explosion gas pressure, leading to the additional separation and propulsion of medium-sized blocks. The consequential impact of these primary fracture networks on the process of rock fragmentation underscores the critical necessity of comprehensively investigating the mechanisms underlying the propagation of cracks induced by stress waves within the medium. Typically, the blast stress wave can be conceptualized as a cylindrical wave, primarily comprising a leading compressive wave followed by a trailing tensile wave [5]. The wave amplitude diminishes as it radiates from the blast center, resulting in varying stress wave strengths experienced by rock masses at different distances from the borehole center. In the presence of numerous joints and faults within rocks, the initial compressive pulse of the blast stress wave might not notably promote defect growth. However, these imperfections will swiftly expand and merge under the influence of the trailing tensile wave. Moreover, repeated instances of blast stress waves can potentially trigger the emergence of large-scale cracks [6,7]. Consequently, a comprehensive inquiry into the dynamic propagation behavior of cracks under blast stress waves becomes paramount.

The challenge of blast stress wave propagation is commonly addressed through the application of a cylindrical wave equation, although its two-dimensional nature complicates the quest for a straightforward general solution. Typically, its resolution is articulated using Hankel functions [8]. Additionally, some researchers have delved into cylindrical wave propagation employing the Laplace transform. Kromm [9] investigated cylindrical wave transmission in the plane stress situation. Historically, Lamb’s study in 1904 [10] presented a systematic exploration of diverse wave propagation modes induced by arbitrary point and line source disturbances on the surface and interior of semi-infinite space. Lamb’s findings notably emphasized the pivotal role played by Rayleigh waves in surface vibration phenomena. This significance extends to the realm of dynamic crack behavior, as these waves’ presence influences the mechanical response due to the existence of two free surfaces. Crack propagation under blast stress waves involves an intricate interplay between stress waves and stationary or propagating cracks [5,11,12]. This phenomenon has been explored by scholars through diverse experimental approaches. Yue [13] utilized the dynamic photoelastic technique to investigate the interaction of blast stress waves with running cracks and studied the effect of P-waves and S-waves on dynamic crack behavior. Yang [12], on the other hand, examined the influence of static stress from different directions on crack dynamic propagation behavior. Investigating the dynamic fracture parameters of four brittle materials subjected to blast loading, Liu’s research [14,15] shed light on essential insights. Wan [16] introduced a rectangle specimen with crack and edge notches (RPCEN), specifically designed to mitigate the influence of reflected waves on crack propagation behavior, thereby facilitating the study of dynamic propagation within blast conditions. Additionally, Yazdani [17] contributed to the discourse by examining crack propagation evolution through a combination of molecular dynamic modeling and experimental study.

Upon encountering a static crack characterized by two tips, stress waves undergo scattering near the closer tip, followed by reflection on the crack surface. This interaction culminates in the creation of a sophisticated superimposed stress field. This stress field exhibits a correlation with the angle of incidence and has prompted the development of certain analytical solutions employing integral transformation techniques [8]. Thau and Tsin’s inquiry [18] focused on the scenario of a plane compressive wave encountering a finite-length crack. Their findings indicated that the dynamic stress intensity attains its peak magnitude upon the passage of the Rayleigh wave. The analytical insights gleaned from these solutions elucidate that, when a P-wave impinges on a crack tip, it can generate a diffraction P-wave, a diffraction S-wave, a head wave, and a Rayleigh wave. These distinct waves exert varied influences on the initiation of further tip behavior, highlighting the critical necessity of identifying the ultimate factor driving crack initiation.

Subsequent to the initiation of a crack tip under the influence of a blast stress wave, the propagation process commences. Freund [19] and B. Merkel [20] discussed fracture and crack propagation in a brittle rock. Hence, a modified model of fracture mechanics was proposed. However, the intricate nature of blast-induced problems renders the attainment of analytical solutions challenging. Unquestionably, crack propagation is intricately linked with the dynamic stress field encompassing the region behind the crack tip in the aftermath of the blast event. The interplay between a propagating crack tip and the dynamic stress field engenders a spectrum of dynamic crack behaviors, including fluctuations in propagation velocity. Typically, crack propagation occurs at a significantly slower pace than the speed of the stress wave, leading to crack arrest either when the dynamic stress wave disengages from the advancing crack or when stress levels prove insufficient to sustain further propagation [21]. The dynamic stress field emerges as a consequence of the perturbation caused by the stress wave’s interaction with the proximal crack tip and its surface. This intricate dynamic prompts a correlation between crack propagation and both the scattering and reflection fields of the stress wave upon encountering the crack. Previous research appears to have largely overlooked the influence of crack length on the intricate dynamics of dynamic crack behavior. However, as per the tenets of elasticity wave theory, the length of the crack should inherently exert a notable impact on the propagation process. In this study, we proposed a numerical model with a borehole and a radial crack to investigate the crack initiation and propagation mechanism induced by blast stress waves. Using sandstone material, we designed a series of twelve distinct models with varying crack lengths to explore the impact of crack length on crack initiation and propagation. Numerical models were established in AUTODYN with consistent mesh characteristics for every model. A triangle pressure curve was used to generate blast waves. Based on the numerical results, we derived some significant conclusions regarding crack propagation under blast waves: crack length critically influences the crack initiation and propagation mechanism. Rayleigh waves contribute significantly to crack propagation in models with long cracks.

## 2. Numerical Study

### 2.1. Dynamic Finite Difference Method

The numerical simulations in this study were conducted using the finite difference method, where each material is represented by one sub-grid. The accelerations $\ddot{u}$ in the *x*-direction and $\ddot{v}$ in the *y*-direction can be calculated by:

(1) $$\left.\begin{array}{c} \ddot{u}=\frac{F_{x}}{m}\\ \ddot{v}=\frac{F_{y}}{m} \end{array}\;\right\}$$

where *F_x_* and *F_y_* are nodal forces in the *x* and *y* directions, respectively, and *m* is mass.

The stress components $\sigma_{x}$, $\sigma_{y}$, and $\tau_{xy}$ can be expressed as [22]:

(2) $$\left.\begin{array}{l} \sigma_{x}=P+S_{x}\\ \sigma_{y}=P+S_{y}\\ \tau_{xy}=S_{xy} \end{array}\right\}$$

where *P* is pressure and *S*_x_, *S*_y_, and *S*_xy_ are deviatoric stresses, which can be calculated by [22]:

(3) $$\left.\begin{array}{l} S_{x}^{n+1}=S_{x}^{n}+2G{\left({\dot{\varepsilon }}_{x}-\frac{1}{3}\dot{e}\right)}^{n+\frac{1}{2}}\;\Delta t\\ S_{y}^{n+1}=S_{y}^{n}+2G{\left({\dot{\varepsilon }}_{y}-\frac{1}{3}\dot{e}\right)}^{n+\frac{1}{2}}\;\Delta t\\ S_{xy}^{n+1}=S_{xy}^{n}+2G{\dot{\varepsilon_{xy}}}^{n+\frac{1}{2}}\;\Delta t \end{array}\right\}$$

where G is the shear modulus and $\dot{e}$ is the volume rate. For two-dimensional problems, $\dot{e}={\dot{\varepsilon }}_{x}+{\dot{\varepsilon }}_{y}$. The material’s equation of state can determine the pressure *P*. For sandstone material, which is a brittleness media, a linear EOS can be used to describe the relationship between the pressure versus density of sandstone material [16]:

(4) $$P=k\cdot \left(\frac{\rho }{\rho_{0}}-1\right)$$

where *k* is the bulk modulus, and $\rho_{0}$ and ρ are initial and current densities. The elastic strength model was applied in stating the relationship between stress and strain for sandstone material, owing to its brittleness characteristics.

### 2.2. Numerical Models

**Figure 2:**
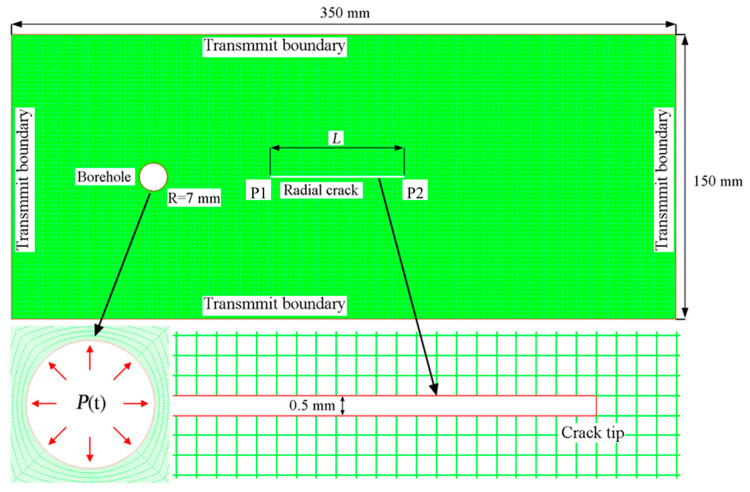
Numerical model.

A numerical model was established in Ansys-AUTODYN code (version 19.2) to investigate the propagation of cracks induced by blast stress waves. The model comprised a rectangular plate measuring 350 mm in length and 150 mm in height. A 7 mm radius hole was designed to generate cylindrical waves. The distance from the hole center to both the left and top edges was 75 mm. On the right side, a radial crack ran along the horizontal symmetrical line of the model, with a length denoted as *L*. The model utilized a structured grid composed entirely of quadrilaterals. To ensure the accuracy of the computational results, the grid was evenly distributed throughout the entire model, consisting of 209,804 elements. The minimum element edge length was 0.4 mm and was located around the boreholes, while the remaining boundary element edges had a length of 0.5 mm, as shown in Figure 2.

### 2.3. Determination of Sandstone Parameters

**Figure 3:**
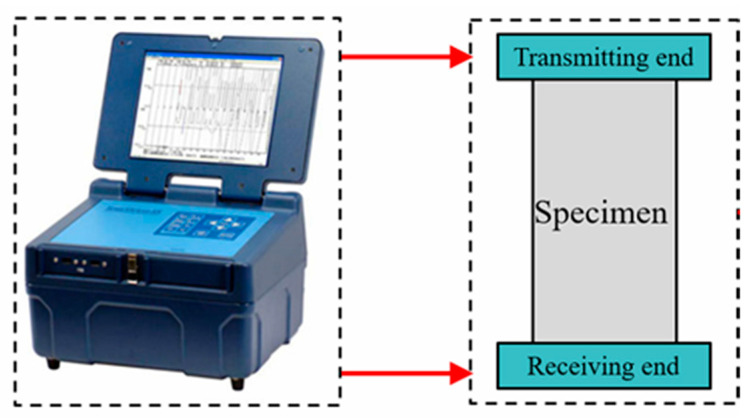
An acoustic velocimeter (Sonic Viewer-SX, Output voltage: 500 V, Pulse width: 6 μs).

The dynamic parameters of the sandstone were measured using an acoustic velocimeter (RSM-SW, Wuhan, China), as depicted in Figure 3. The velocimeter consisted of two piezoelectric ceramic ends: the transmitting end, which generated wave signals, and the receiving end, which was responsible for signal reception. By analyzing the wave traveling time through the specimen and its geometry configuration, the velocities of the P-wave and S-wave could be accurately calculated.

Substituting the speeds of the P-wave and S-wave, cs and cd, into Equations (5) and (6), the dynamic elastic modulus and Poisson’s ratio can be acquired.

(5) $$E_{d}=\frac{\rho c_{s}{}^{2}\left(3c_{d}{}^{2}-4c_{s}{}^{2}\right)}{c_{d}{}^{2}-c_{s}{}^{2}}$$

(6) $$v_{d}=\frac{c_{d}{}^{2}-2c_{s}{}^{2}}{2c_{d}{}^{2}-2c_{s}{}^{2}}$$

where *ρ* is the density of the sandstone, $E_{d}$ is the dynamic elastic modulus, and *v_d_* is the Poisson’s ratio. The speed of the Rayleigh wave of the sandstone material can be calculated by [8]:

(7) $$c_{R}=\frac{0.862+1.14\;v_{d}}{1+v_{d}}c_{S}$$

where $c_{R}$ is the Rayleigh wave speed. All the dynamic parameters of the sandstone material are listed in Table 1.

## 3. The Interaction between Stress Wave and Crack

**Figure 4:**
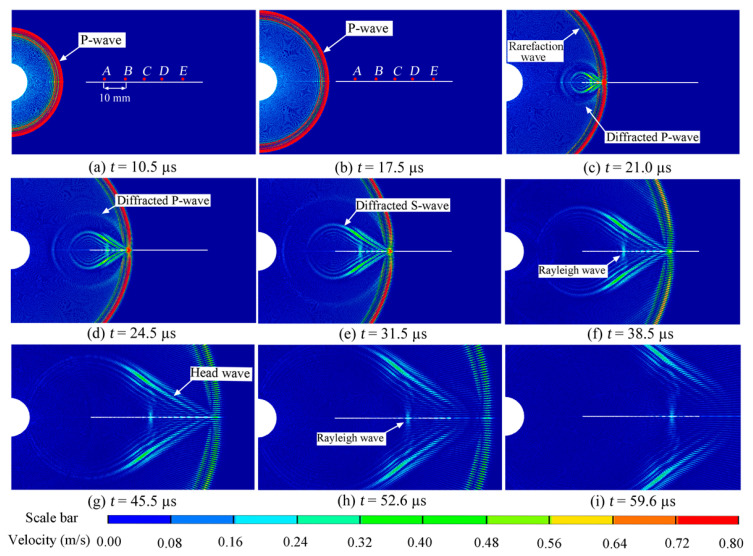
Stress wave-induced particle velocity vectors.

To effectively demonstrate the interaction between the stress wave and crack, a short pulse is necessary. Otherwise, the interaction process would be prolonged and susceptible to the influences of rarefaction waves and reflected waves. Hence, a rectangular compressive pulse with an amplitude of 50.0 MPa and a duration of 0.1 µs was applied to the blasthole wall. The crack length was set at 60 mm, and the distance from the left tip of the crack to the blasthole center was 40 mm. Employing the model represented in Figure 2, a series of numerical simulations were meticulously undertaken. The simulation outcomes, capturing the patterns of the particle velocity vectors induced by the transient rectangular pulse, are distinctly presented in Figure 4.

A compressive P-wave is evident in Figure 4a,b, accompanied by a rarefaction P-wave. When the P-wave encountered the left tip of the crack, it gave rise to a diffracted P-wave and diffracted S-wave, as shown in Figure 4c, with their wavefronts forming circles centered at the crack’s left tip. As the diffracted P-wave and S-wave traveled, their amplitudes gradually attenuated, leading to the observation of two wavefronts: a diffracted P-wave and diffracted S-wave, as depicted in Figure 4d,e, respectively. Additionally, apart from the diffracted P-wave and S-wave, two other types of waves were present. The first was the head wave, which represented the reflected S-wave as the P-wave propagated along the crack surface. The second was the Rayleigh wave, which traveled along the crack surface and induced significant displacement in the vertical direction.

**Figure 5:**
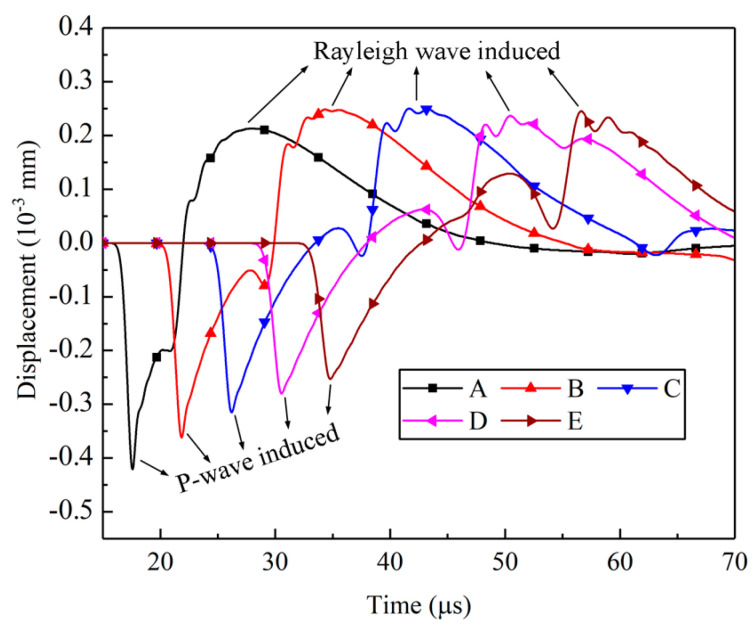
Displacements along the crack surface.

These waves were capable of instigating vibrations across the crack surface, each with distinct amplitudes. For a comprehensive analysis of the surface displacements, a set of five gauge points (A, B, C, D, and E) was strategically located at 10 mm intervals along the upper crack surface. These gauge points were instrumental in capturing the temporal evolution of displacements in the y-direction, as clearly depicted in Figure 5. Notably, the displacement history curves exhibit a discernible bifurcation into two distinct stages: negative and positive. During the negative stage, the displacements manifested as negative values, signifying a tendency for the crack to contract. This closure phenomenon was chiefly driven by the compressive pulse, particularly the P-wave, characterized by the highest propagation velocity. However, it is important to note that the attenuation of the P-wave induced a noticeable decline in the peak displacements of the five designated points (A, B, C, D, and E) within the negative stage. This diminution was particularly prominent with an increasing distance from the crack’s left tip.

**Figure 6:**
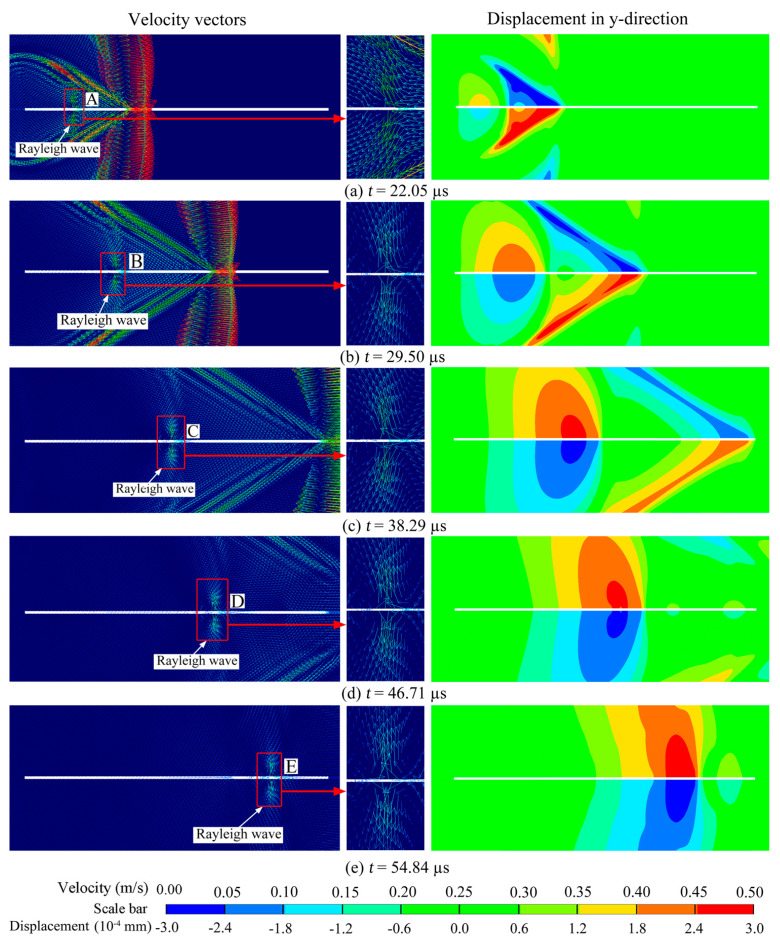
Velocity vectors on crack surface induced by Rayleigh wave.

In Figure 5, all the curves exhibit a non-smooth transformation from negative to positive values, influenced by the rarefaction P-wave, the head-wave, and the diffracted S-wave. These three waves traveled behind the P-wave but in front of the Rayleigh wave, as depicted in Figure 6.

During the positive stage, the peak displacements at points A, B, C, D, and E in Figure 5 show minimal changes, which differ significantly from the negative stage induced by the P waves. Figure 6 displays the particle velocity vectors caused by the Rayleigh waves and the corresponding y-direction displacements at points A, B, C, D, and E. This observation suggests that the amplitude attenuation of the Rayleigh waves during their travel along the crack surface was minimal, which aligns with theoretical findings by Achenbach [8].

Conversely, the amplitude of the P-wave or the diffracted P-wave diminished rapidly. In contrast, the amplitude attenuation of the Rayleigh wave was comparatively slight, underscoring its pivotal role in initiating and propagating cracks at the distant tip. It is worth highlighting that, in scenarios where the far tip of the crack is located at a substantial distance from the borehole, the P-wave experiences pronounced attenuation, rendering it less effective in triggering initiation at the far tip.

## 4. Crack Dynamic Propagation under Blast Stress Wave

When simulating material failure, there are many failure criteria, such as the MTS criterion, which can accurately simulate the direction of crack initiation [23,24]. In this study, the linear propagation of the crack was analyzed. Therefore, the principal stress failure criterion presented in Equation (8) was utilized to predict the material’s response [25]. According to this criterion, failure occurred when the principal stress $\sigma_{1}$ exceeded the dynamic tensile strength $\sigma_{T}$.

(8) $$\sigma_{1}\geq \sigma_{T}$$

**Figure 7:**
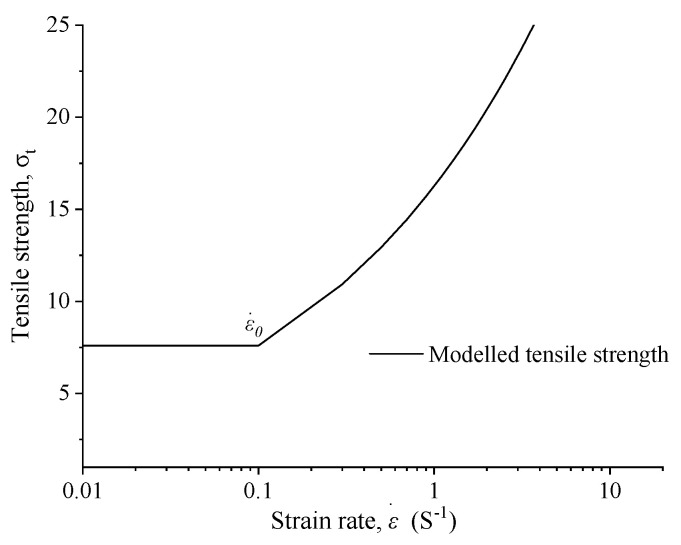
Illustration of the bilinear relationship.

As commonly recognized, the dynamic tensile strength $\sigma_{T}$ exhibited a significant rate effect, as demonstrated by numerous experimental findings. In this paper, Equation (9) was used to characterize the correlation between the dynamic tensile strength of the sandstone and the strain rate [26,27,28].

(9) $$\sigma_{T}=\left\{\begin{array}{cc} \sigma_{\mathrm{st}} & \dot{\varepsilon }\leq {\dot{\varepsilon }}_{0}\\ \exp\left(0.33\;\ln\left(\frac{\dot{\varepsilon }}{{\dot{\varepsilon }}_{0}}\right)\right)\sigma_{\mathrm{st}} & \dot{\varepsilon }>{\dot{\varepsilon }}_{0} \end{array}\right.$$

where $\sigma_{\mathrm{st}}$ is the static tensile strength of the sandstone, $\dot{\varepsilon }$ is the equivalent strain rate, and ${\dot{\varepsilon }}_{0}$ is the critical strain rate. It essentially describes a bilinear relationship between the strain rate and tensile strength, as illustrated in Figure 7.

**Figure 8:**
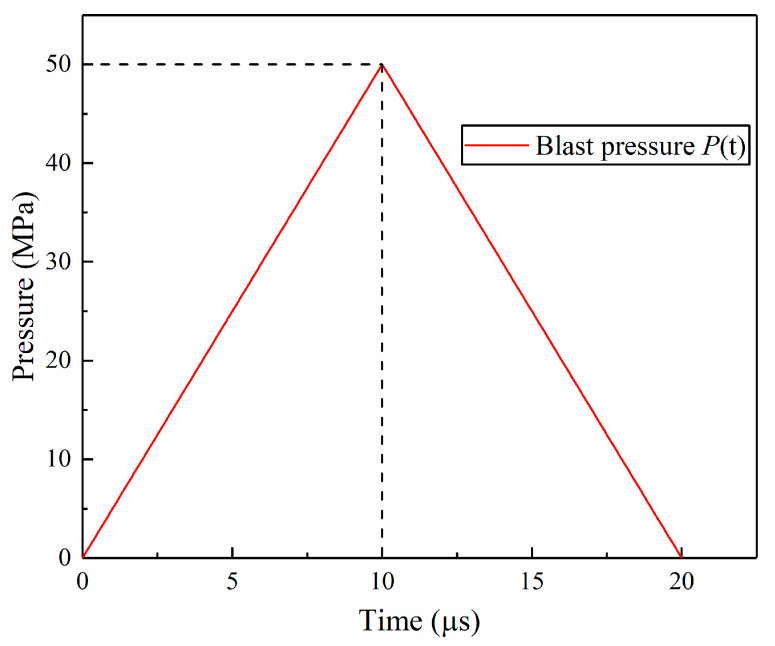
Loading curve.

In the context of a solitary blasting event, the loading profile engendered by an electrical detonator can be approximated by a triangular curve, as illustrated in Figure 8. This triangular loading curve spans a duration of 20 µs, reaching a peak value of 50 MPa. Although there might exist minor deviations from the actual blast loading sequence, this approximation is advantageous, as it streamlines the calculation process and conveniently mitigates the impact of certain less influential variables.

In this simulation, the loading curve P(t) was applied on the borehole wall, as shown in Figure 8, to investigate the crack initiation and propagation behavior for models with varying crack lengths. The crack possessed a width of 0.5 mm, and its left tip remained anchored at a distance of 10 mm from the center of the borehole. To systematically investigate its behavior, the crack’s length spanned from 10 mm to 120 mm, with intervals of 10 mm, thereby engendering the formulation of a total of 12 distinct models.

### 4.1. The Waves around Crack Surfaces and the Wave-Induced Displacements

**Figure 9:**
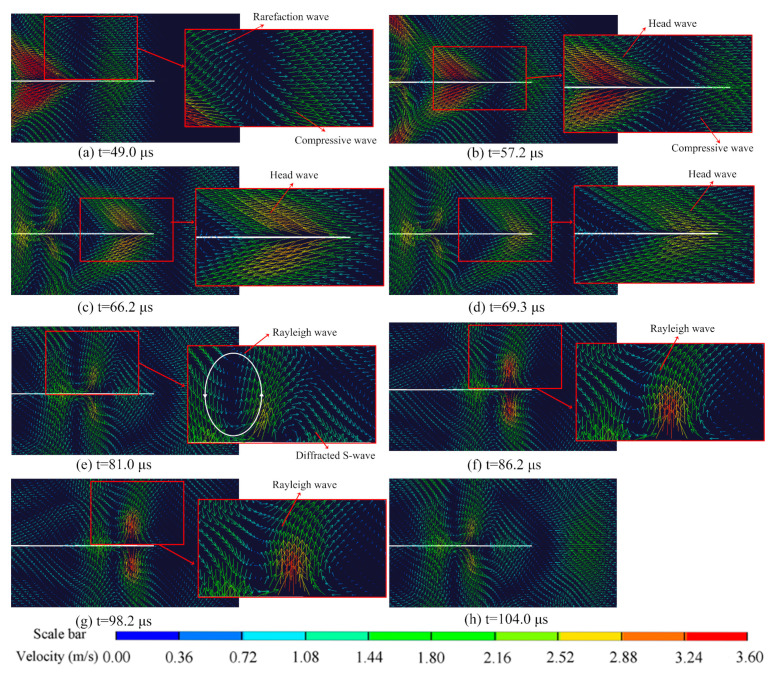
Particle velocity vectors near the crack surface.

**Figure 10:**
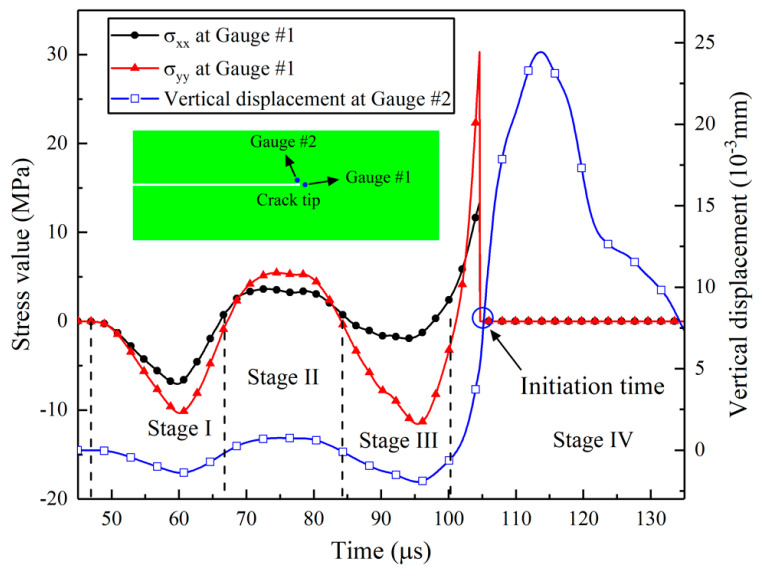
Stress history curves at the crack tip.

The model with a crack length of 110 mm was selected as an example to illustrate the influence of various waves in the crack initiation process. Figure 9 displays the particle velocity vectors near the crack at different moments. The P-wave, as the leading compressive pulse, possessed the highest traveling speed, as evident in Figure 8a,b. Observing the velocity vectors, it becomes apparent that the crack closed under compression. This phenomenon occurred because the crack surface was free, leading the particles on the surface to move outward when subjected to compression. This observation is supported by the displacement of gauge 2 at the crack tip in stage I, as shown in Figure 10.

The rarefaction wave, or tailing tensile pulse, followed the compressive pulse, as depicted in Figure 9a. Additionally, the head wave induced by the P-wave propagating along the crack surface is visible in Figure 9b. Under the combined influence of the rarefaction wave and head wave, the crack began to open, as indicated by the motion of the particles in Figure 9c,d. In stage II of Figure 9, both the stresses σx and σy are positive, indicating that the crack tip was subjected to biaxial tensile stresses, and the corresponding vertical displacement is positive. However, the displacement remained too small to cause the crack to fully open.

Following the head wave, the diffracted S-wave becomes apparent in Figure 9e, inducing particle motion perpendicular to the wave’s travel direction and causing the crack tip to close. Another notable characteristic in stage III was the more pronounced change in σy compared to σx due to the shear effect. Additionally, the Rayleigh wave, as observed in Figure 9e,f trailing the diffracted S-wave, caused the particles to move in an elliptical pattern. As depicted in Figure 9f–h, as the Rayleigh wave traveled along the crack surface, it induced significant lateral movement in the zone near the crack tip, resulting in relatively large velocity vectors. Consequently, the Rayleigh wave induced a more prominent open displacement, and during the period when the crack tip was under biaxial tensile stress, similar to the S-wave, σ_y_ experienced a more considerable increase than σ_x_.

### 4.2. The Formation of Rayleigh Wave

**Figure 11:**
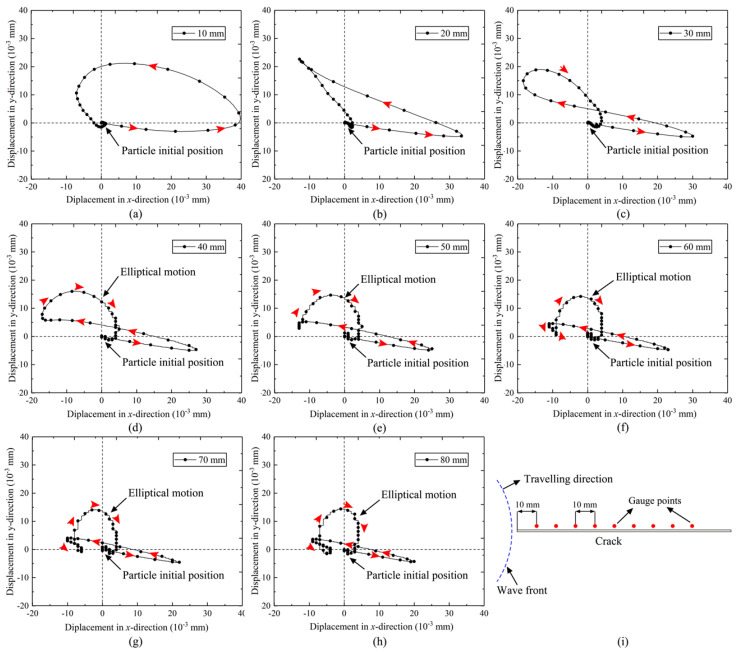
Motion paths of particles along the crack’s upper surface ((**a**–**h**), motion paths of particles; (**i**), display of measurement point layout).

The Rayleigh wave, known to be a product of the diffracted P-wave and S-wave, induced elliptical motion of the particles near the crack surface, leading to a much more significant vertical displacement, as shown in Figure 10. Eight gauge points were strategically positioned along the crack’s upper surface at intervals of 10 mm to elucidate the formation of the Rayleigh wave in this study, as depicted in Figure 11i, to record the displacements in the x-direction and y-direction. The results presented in Figure 11 illustrate the particle trajectories along the crack face during wave grazing.

The figures demonstrate that the particles distributed along the crack’s upper surface underwent different motion paths. In Figure 11a,b, no elliptical movement is observed, signifying the absence of the Rayleigh wave near the left tip of the crack. Nonetheless, a discernible contrast in motion patterns was evident, characterized by the shared trait of anti-clockwise motion. This behavior arose from the interplay between the leading compressive pulse of the diffracted P-wave and the arrival of the diffracted wave itself, resulting in their superposition and consequent anti-clockwise particle motion. With the progressive increment in the distance from the crack’s left tip, the temporal overlap between the diffracted P-wave and S-wave transitioned from aligning with the stage of action of the compressive P-wave to aligning with the stage of action of the rarefaction wave. Consequently, the particle trajectory changed from anti-clockwise to clockwise movement, as depicted in Figure 11c–e. The tendency of ellipse movement becomes evident in Figure 11e.

As the distance continued to increase, ellipse motion is observed in Figure 11f–h, indicating the appearance of a Rayleigh wave. This phenomenon results from the superposition of the diffracted S-wave and the later-acting stage of the rarefaction wave. Notably, the Rayleigh wave exhibited a distinct characteristic: the length of the long axis of the ellipses in Figure 11f–h is longer than that of the short axis.

### 4.3. Crack Propagation

#### 4.3.1. Particle Motion around a Moving Crack Tip

**Figure 12:**
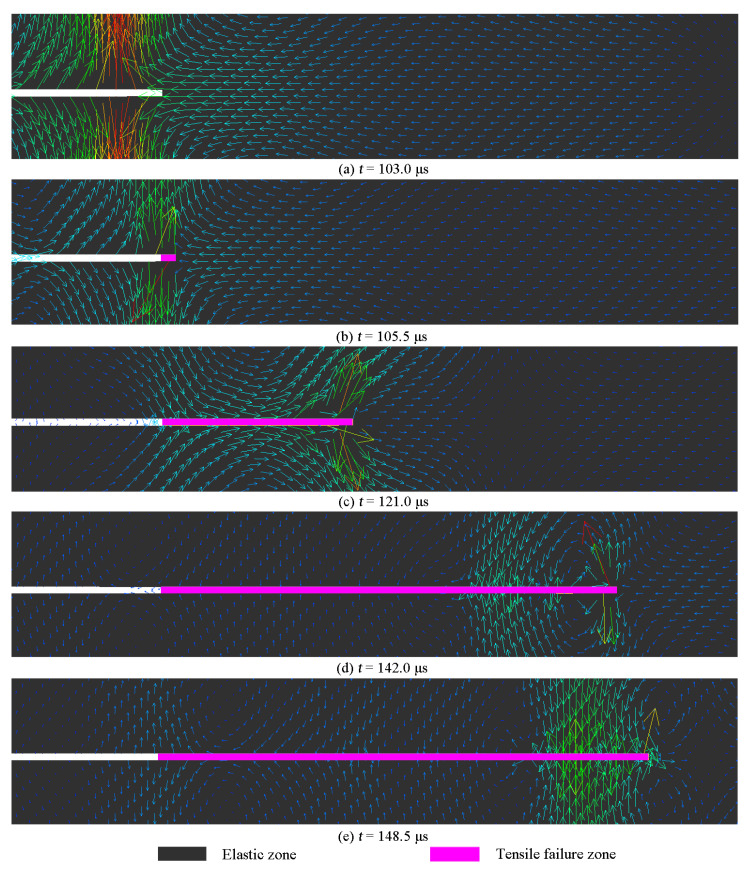
Velocity vectors around a running crack tip.

As depicted in Figure 12a, the Rayleigh wave induced a substantial displacement in the y-axis direction, subjecting the crack tip to significant tensile stress. Consequently, the crack was compelled to propagate, giving rise to the formation of a new crack surface, as illustrated in Figure 12b. As the Rayleigh wave continued propagating along the newly formed crack surface, the crack tip advanced further, as demonstrated in Figure 12c. Due to the particles’ elliptical motion, the latter part of the Rayleigh wave caused the crack to close, as shown in Figure 12d. Eventually, when the Rayleigh wave reached the crack tip, the crack arrested, as presented in Figure 12e.

#### 4.3.2. The Effect of Crack Length

**Figure 13:**
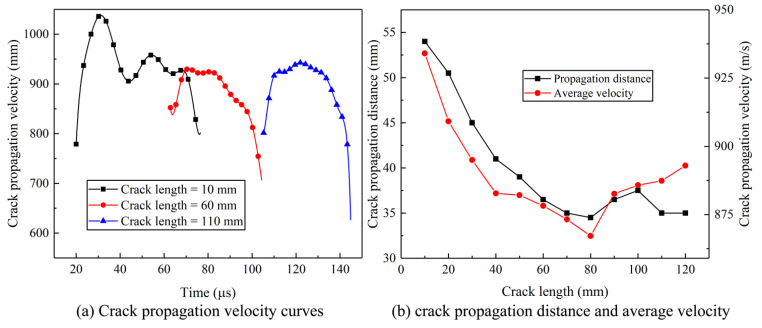
The continuous decrease in crack propagation velocity and distance of different crack lengths until stable variation characteristics.

Three typical crack lengths (10 mm, 60 mm, and 110 mm) were selected to investigate the effect of crack length on the crack propagation behavior in detail, and their velocity functions versus time are plotted in Figure 13a. In the case of a 10 mm crack length, the crack velocities underwent an initial rapid augmentation, followed by a subsequent decline, temporary fluctuations, and finally, a rapid deceleration until crack arrest. This pattern was consistently observed across varying crack lengths: an initial velocity increase, followed by a phase of relatively stable velocities, culminating in a rapid deceleration leading to crack arrest. It is noteworthy that shorter crack lengths exhibited substantially elevated peak velocities, with the corresponding curves manifesting heightened vibration characteristics. These pronounced vibrations stemmed from intricate stress superpositions occurring at the advancing crack tip within such configurations. In contrast, longer crack lengths were predominantly influenced by the Rayleigh wave, which imparted consistent attributes to the velocity curves. Average velocities and propagation distances for models with different crack lengths were obtained to analyze the concrete influence of crack length on crack propagation, as shown in Figure 13b. Evidently, the propagation distances exhibited a gradual decay as the crack length increased, ultimately attaining a state of stabilization when the crack length achieved a certain extent. This characteristic was coupled with a corresponding trend in average velocities, wherein a gradual decline was evident in the initial stages. Notably, at a critical crack length of approximately 80 mm, the average velocity reached its nadir, plausibly attributed to the influence of the S-wave. As crack length continued to increase, the distinctive influences of the shear wave and Rayleigh wave came to the fore, culminating in a gradual elevation of the average crack velocity. This phenomenon underscores the growing prominence of these waves in shaping the behavior of crack propagation.

## 5. Discussion

The stress field localized at the crack tip assumed a pivotal role in governing the intricate phases of crack initiation, propagation, and eventual arrest. This field is inherently subject to the dynamic response exhibited by the P-waves and S-waves disseminating within the medium and the Rayleigh waves traversing along the crack surface. As meticulously elucidated within the confines of this paper, the length of the crack exerted a discernible influence upon the juxtaposed stress histories of P-waves, S-waves, and Rayleigh waves at the crack tip. Consequently, this interplay yielded a panorama of varied dynamic propagation behaviors. The movement of a short crack tip followed the pattern shown in Figure 11a. In the preliminary stages, the crack underwent closure due to the compressive P-wave. Subsequent to this, as the tensile rarefaction wave and S-wave reached the scene, a substantial opening displacement ensued at the crack tip. This pivotal event marks the instigation of crack initiation, giving rise to the formation of a nascent fracture surface. This event holds considerable sway over the subsequent patterns of crack propagation. Notably, the particle velocity attained its zenith during the initial phase, a direct consequence of the amplitude of the stress waves. This augmented particle velocity begot an accelerated crack-opening process. As a result, in the model characterized by a 10 mm crack length, the velocities of crack propagation during the primary phase were notably superior in comparison to later time intervals. This tendency was likewise evident in the case of a 20 mm crack length.

**Figure 14:**
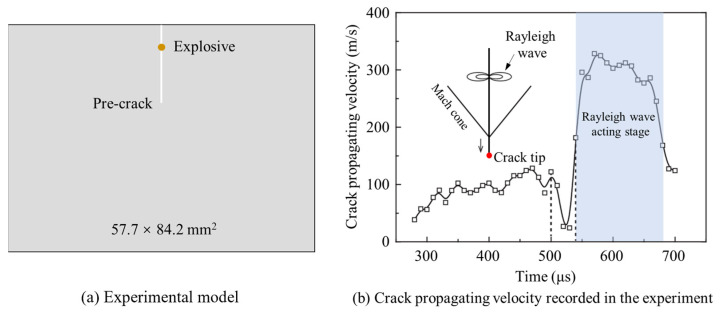
The explosion experiment and the crack-propagating velocity were recorded in the experiment [5].

As the length of the crack increased, the Rayleigh wave became visible on the surface. By comparing the impacts of the P-wave and Rayleigh wave on the crack tip, it was clear that the Rayleigh wave caused significantly greater opening displacement than the P-wave (see Figure 11d–i). Models with longer crack lengths are governed by Rayleigh waves, which gradually open the crack as it passes the running crack tip, sustaining the process of crack fracturing. After the peak value of the Rayleigh wave passes, the crack tip tends to close due to the falling edge of the Rayleigh wave, leading to crack arrest. The Rayleigh wave does not noticeably diminish with its traveling distance on the crack surface, inducing similar propagation behaviors, as seen in the propagation velocity curves of models with 60 mm and 100 mm crack lengths shown in Figure 11a. Similar results were observed for other long crack-length models. These findings highlight the significant role of Rayleigh waves in promoting crack propagation, which is supported by Yue’s explosion experimental results [5], as shown in Figure 14a. The experiment recorded crack propagation velocities under blast stress waves, as shown in Figure 14b, and it was evident that the crack-propagating velocity increased notably when the Rayleigh wave reached the crack tip, thus corroborating the numerical results in this paper.

## 6. Conclusions

This paper demonstrated a numerical model encompassing a borehole and a radial crack, conceived to delve into the intricate mechanisms underpinning the crack initiation and propagation incited by blast stress waves. Twelve distinct models were devised utilizing sandstone, each characterized by varied crack lengths, enabling an exploration of the nexus between crack length and the initiation propagation dynamics. The research outcomes are encapsulated as follows:

(1) During the propagation process of stress waves, when encountering a crack, they will first generate a diffused P-wave and a diffused S-wave, and then continue to propagate along the crack, generating a head wave and a Rayleigh wave.

(2) The localized stress fields at crack tips govern crack initiation and propagation.

(3) In cases of shorter crack lengths, crack initiation and propagation are instigated by the confluence of the P-wave and S-wave. Conversely, in instances of elongated crack lengths, the Rayleigh wave assumes primacy, orchestrating crack initiation and propagation and thereby conferring uniformity to the propagation behaviors.

(4) As the crack length increases, the crack propagation velocity and distance exhibit a decreasing characteristic and eventually stabilize.

(5) When Rayleigh waves exist on the surface of a long crack, the important role of Rayleigh waves in promoting crack propagation is experimentally verified.

It is important to underscore that the aforementioned conclusions stem from the numerical-model-formulated mode I crack. Subsequent research endeavors should be directed toward scrutinizing and elucidating the propagation dynamics of mixed-mode cracks.

## Figures and Tables

**Table 1:** Parameters of sandstone used in this study.

Material	Density /kg/m^3^	Bulk Modulus/GPa	Elastic Modulus /GPa	Possion Ratio v*_d_*	c*_d_* /m/s	c*_s_* /m/s	c*_R_* /m/s
Sandstone	2380	9.462	17.60	0.19	2549.4	1459.2	1322.6

## Data Availability

Data are contained within the article.
